# Fungal Infection in HIV-Infected Patients—Is It Still a Challenge?

**DOI:** 10.3390/jcm14238570

**Published:** 2025-12-03

**Authors:** Martyna Biała, Natalia Słabisz, Brygida Knysz

**Affiliations:** 1 Department of Infectious Diseases, Liver Diseases and Acquired Immune Deficiences, Faculty of Medicine, Wroclaw Medical University, 51-149 Wroclaw, Poland; 2 Department of Preclinical Sciences, Pharmacology and Medical Diagnostics, Faculty of Medicine, Wroclaw University of Science and Technology, 50-370 Wroclaw, Poland; 3 Department of Laboratory Diagnostics, 4th Military Clinical Hospital, 53-114 Wroclaw, Poland

**Keywords:** HIV, fungal infection, antifungal resistance

## Abstract

Fungi are one of the major causes of opportunistic infections in people living with HIV/AIDS. *Pneumocystis jirovecii*, *Cryptococcus neoformans*, *Histoplasma capsulatum*, and *Candida* spp. are especially more likely to affect HIV-infected individuals. Introduction and broad use of antiretroviral therapy have led to a significant decrease in invasive fungal infections in HIV-positive patients. Still, in untreated/abandoned HIV-infected individuals or patients with poor adherence to antiretroviral therapy, fungal infections may lead to severe clinical complications and death. In recent years, data have shown a growing number of immunocompromised patients due to malignancies, immunosuppressive therapies, and transplantations in the general population, and the number of susceptible individuals to fungal infections is increasing. Moreover, rising antifungal resistance is a serious threat to public health. This article provides an overview of common fungal infections in patients with HIV and discusses the changes in epidemiology and etiology, as well as current therapeutic challenges.

## 1. Introduction

According to World Health Organization (WHO) data, nearly 40.8 million people were living with HIV at the end of 2024, and 1.3 million people were newly diagnosed with this infection in 2024 [1]. Moreover, globally, 630,000 people died from HIV-related diseases in 2024 [1]. HIV-positive individuals are at risk of opportunistic infections in the advanced stage of disease, defined as CD4 T cell counts <200 cells/mm^3^. Introduction and broad use of antiretroviral therapy have significantly decreased the risk of opportunistic infections and reduced mortality rates in people living with HIV [2]. A study showed that the life expectancy of patients with HIV who were diagnosed early and started antiretroviral therapy is only a few years lower than that of the general population [3]. However, individuals with untreated or poorly controlled HIV infection remain at a significantly increased risk of developing opportunistic infections caused by bacteria, viruses, fungi, or protozoa, and their mortality rates remain high. Moreover, in recent years, data have recorded a growing number of immunocompromised patients due to malignancies, immunosuppressive therapies, and transplantations in the general population, and the number of susceptible individuals to fungal infections is increasing [4,5]. Fungal diseases are common in HIV-positive individuals and can occur in the early stage of HIV infection and in the advanced stage of HIV as serious systemic fungal infections caused by *Pneumocystis jirovecii*, *Cryptococcus neoformans*, *Histoplasma capsulatum*, and other fungal species. In 2022, the World Health Organization (WHO) released the first fungal priority pathogen list, classifying 19 fungal species into three priority levels—critical, high, and medium—to guide research priorities (Table 1) [6]. The ranking was established through a multi-criteria decision analysis that took into account both research and development requirements as well as the pathogens’ perceived public health significance. However, it is essential to emphasize the regional differences in the incidence of fungal infections, e.g., *Talaromyces marneffei*, a dimorphic fungus found endemically in tropical and subtropical Asia, represents a significant risk for individuals with impaired cellular immunity, such as those living with HIV or transplant recipients. Still, in other regions, it is rare in clinical practice, but it may be imported by potentially vulnerable travelers who have visited areas of the endemic region [7]. Some of the species from the WHO fungal priority list are common in patients with HIV regardless of the geographical region.

In this article, we provide an overview of common fungal infection in patients with HIV and discuss the changes in epidemiology and etiology as well as current therapeutic challenges.

## 2. Common Fungal Infections in Patients with HIV

### Candida Infections

Although more than 160 *Candida* species exist, only about 20 are known to lead to disease in humans and over 90% of invasive candidiasis is caused by the five most common pathogens: *C. albicans*, *C. glabrata*, *C. tropicalis*, *C. parapsilosis*, and *C. krusei* [8,9]. However, the relative frequency of these species differs according to factors such as age, affected population, previous exposure to antifungal drugs, and geographic location [9]. Infections caused by the *Candida* species can manifest in a wide spectrum, ranging from localized mucosal infections to systemic disease involving multiple organ failure [8]. While the *Candida* species are normally part of the human skin, the gastrointestinal and genitourinary microbiota, they can become pathogenic, and the host’s immune status plays a crucial role in determining the type and severity of *Candida* infection [8]. Mild infections typically result from excessive local growth on mucosal surfaces, such as oral thrush or vaginal candidiasis, often due to alterations in one’s own microbiota, but in immunocompromised patients *Candida species* can cause life-threatening, invasive infections.

Oropharyngeal and esophageal candidiasis frequently occur in individuals with HIV [10]. The presence of oropharyngeal or esophageal candidiasis is considered as a marker of immune suppression and is most often seen in individuals with CD4 T cell counts below 200 cells/mm^3^, with esophageal involvement generally associated with even lower CD4 levels than oropharyngeal disease [10,11,12]. Conversely, vulvovaginal candidiasis— first episode or recurrent—is common among healthy adults and is not indicative of HIV infection [10]. Main clinical forms of oropharyngeal candidiasis include pseudomembranous (exudative), chronic atrophic stomatitis-denture stomatitis, angular cheilitis, chronic hyperplastic candidiasis (*Candida* leukoplakia), and midline glossitis-acute atrophic stomatitis [13]. Oropharyngeal candidiasis usually presents as painless, creamy white, plaque-like lesions that may appear on the buccal mucosa, hard or soft palate, gums, tongue, and oropharynx [10,13]. These lesions can often be removed by scraping. Less frequently, erythematous areas without white plaques may be observed on the anterior or posterior palate or diffuse across the tongue [10,13]. It is estimated that oropharyngeal candidiasis affects about 90% of people living with HIV once CD4 T cell counts decline below 200 cells/mm^3^ [14,15,16,17]. Its prevalence among HIV-infected individuals shows considerable geographic differences, ranging from 17.8% to 44.2% in India, 23.6% in Nepal, 31.6% in Mexico, and up to 79.4% across different African countries [17,18,19,20,21,22,23,24]. Studies assessing the global changing pattern of the oral manifestations of HIV showed that the greatest reported prevalence of oral candidiasis was in Africa (51%) and Asia (39%); furthermore, the average prevalence in Europe and in the USA was similar—28% and 30%, respectively [25]. Participants receiving antiretroviral therapy showed a lower overall prevalence of oropharyngeal candidiasis (26.2%) compared with those not on ART (39%), although the difference was not considerable [25]. Oropharyngeal candidiasis is commonly observed in individuals with various underlying health conditions and risk factors (e.g., people with diabetes; those using steroid inhalers or long-term antibiotics; denture wearers; smokers) and is not exclusive to HIV; this may explain why there is no considerable difference [25].

Previous studies assessing prevalence of esophageal candidiasis among patients with HIV showed differences between geographical regions, with results ranging from 6% in France, 15.8% in a Pan-European study, 9.8% in Japan, and 12% in sub-Saharan Africa [26,27,28,29]. However, little up-to-date data about the prevalence of esophageal candidiasis among patients with HIV is available.

Invasive candidiasis seems to be a rare complication among treated patients with HIV. Retrospective study from the French Guiana revealed that among 274 HIV-infected individuals with invasive fungal disease, 31 patients (11.3%) had positive blood cultures for *Candida* spp. (including 15 cases of *Candida albicans* and 16 cases of *Candida non-albicans*) [30]. Seven patients were treated in intensive care units, and overall, five individuals died within a year [30]. Parra–Giraldo CM, et al. presented three cases of emerging fungus *C. auris* infection identified in patients with known risk factors for fungal disease—one in an intensive care unit (ICU) patient, one with lymphoma, and one with HIV [31]. All of these patients had a history of prior antibiotic use, and the cases showed no epidemiological connection to each other [31]. Population-based active surveillance for culture-confirmed candidemia in 2017–2021 including 10 sites of the United States showed that diabetes was the most frequent underlying condition, accounting for 36.2% of cases, with its prevalence rising from 32.1% in 2017 to 38.0% in 2021 [32]. The frequencies of other underlying conditions remained relatively consistent throughout the study period, with HIV accounting for 1.9% of all cases (2.4% (2017), 2.3% (2018), 1.8% (2019), 1.9% (2020) and 1.4% (2021)) [32]. Main risk factors associated with invasive candidiasis/candidemia include neutropenia, central venous catheters, prolonged ICU stays, mechanical ventilation, age, broad-spectrum antimicrobials use, uncontrolled diabetes mellitus, solid organ transplants, intravenous drug use, parenteral nutrition, and abdominal surgery [33,34]. Invasive candidiasis and candidemia usually develop in individuals with one or more risk factors, most commonly among those who are immunocompromised or critically ill. Conventional direct microscopy and culture-based techniques remain the primary methods for diagnosing both superficial and invasive *Candida* infections [35]. While biomarkers and molecular tests can be useful in specific clinical contexts, solid evidence supporting their broader application is still lacking [35].

## 3. Therapeutic Challenge of Treating *Candida* Infections in Patients with HIV

A growing number of patients are developing *Candida* infections (non-invasive and invasive) that are challenging to manage due to rising antifungal resistance. A systematic review and metanalysis assessed the occurrence of drug-resistant *Candida* species caused oral infections in HIV-positive patients and showed that pooled prevalence of resistance to the antifungal medicines was as follows: ketoconazole (25.5%), fluconazole (24.8%), 5-flucytosine (22.9%), itraconazole (20.0%), voriconazole (20.0%), miconazole (15.0%), clotrimazole (13.4%), nystatin (4.9%,), amphotericin B (2.9%), and caspofungin (0.1%) [36]. Another study revealed that among seventy-five oral *Candida* isolates obtained from HIV-positive participants, eighteen (24%) were resistant to fluconazole, four (5.3%) were susceptible-dose-dependent, and 53 (70.7%) were classified as susceptible [23]. Tamai IA, et al. showed that 32%, 28%, and 14% of oral *Candida* isolates were resistant to fluconazole, ketoconazole and clotrimazole, respectively [37]. There are limited data about resistant isolates causing bloodstream infections in HIV-infected individuals. However, the extensive use of antifungal medicines has led to the global decrease in azole-derivative susceptibility of *Candida* isolates, and the emergence of multidrug-resistant *C. auris* and *C. glabrata* [38]. *C. glabrata*, frequently resistant to azole-derivatives, marks the emergence of the most common invasive non-*C. albicans* species reported to the SENTRY surveillance program across North America, Europe, and the Asia-Pacific region, except for Latin America, where *C. parapsilosis* and *Candida tropicalis* were more prevalent [39]. Moreover, azole-derivative resistant *C. parapsilosis* has also emerged as a global concern, driven by the widespread dissemination of resistant clonal strains, with multiple outbreaks documented in Europe, the United States, and Brazil [40]. A recent meta-analysis of 79 studies found that fluconazole resistance among *C. parapsilosis* isolates rose from 11.6% to 36.7% between 2016 and 2022 [41]. Moreover, the number of susceptible individuals to fungal infections is increasing in general populations. This trend contributes to higher healthcare use, greater economic costs, and increased mortality. In addition, the emergence of pathogens such as *Candida auris* (*Candidozyma auris*) and fluconazole-resistant *Candida parapsilosis* represents a serious global health concern, especially in hospital settings where transmission can occur easily. These findings highlight the need to monitor the growing burden of the antifungal resistance of *Candida* isolates in both HIV-infected and seronegative counterparts. Treatment of mucocutaneous candidiasis is dominated by the azole-derivatives agents used topically or systemically. Echinocandins are recommended as the first-line therapy for candidaemia and most types of invasive candidiasis, except for infections involving the CNS or ocular infections [35]. Other treatment options include liposomal amphotericin B (sometimes used with flucytosine) and fluconazole, though potential azole-derivative resistance must be considered [35].

## 4. *Pneumocytstis jirovecii* in Patients with HIV

The occurrence of *Pneumocystis jirovecii* pneumonia (PJP) has significantly decreased, mainly because of effective antiretroviral therapy and prophylaxis use. However, it continues to be a major opportunistic infection among individuals with HIV and low CD4 T cell counts, particularly those who are unaware of their infection or are untreated/abandoned. Wolff AJ, et al. assessed the change in pulmonary manifestations in patients with HIV since the introduction of highly active antiretroviral therapy and compared clinical data from 1993 to 1995 and 1997 to 2000 [42]. This study showed that PJP was less common in antiretroviral therapy era. Another study showed that incidence rates of PJP among patients with HIV in the United States declined significantly over time from 2000 to 2010 from 0.92 per 100 person-years to 0.39 per 100 person-years [43]. In Spain, the incidence of PJP lowered significantly from 13.4 cases per 1000 patients-year in 2000 to 3.3 cases per 1000 patients-year in 2013 [44]. A systematic review and meta-analysis assessed global prevalence, mortality, and main characteristics of HIV-associated pneumocystosis from 2000 to 2022 and revealed that the pooled prevalence of HIV-associated pneumocystosis was 35.4%, whereas the pooled frequency of PJP among HIV-negative counterparts was 10.16% [45]. Moreover, HIV-positive individuals were almost 12 times more vulnerable to PJP than the HIV-negative patients [45]. Analysis showed also that mortality among people living with HIV diagnosed with PJP was 52% higher than in non-PJP patients [45]. Danish researchers showed that among 4882 HIV-infected individuals, they observed 336 PJP cases, but for the time period 2016–2021, compared to 1995–1999, PJP risk declined by over 90% after the first year of HIV diagnosis, and by over 40% in the first year, if baseline CD4 T cell count was <200 cells/µL [46]. The risk of developed PJP stayed high if the CD4 T cell count was 100 to <200 cells/during the first year of antiretroviral therapy, despite a suppressed viral load [46]. The Danish HIV cohort study revealed that PJP continues to pose a serious concern among individuals diagnosed with HIV especially in late-presenters and highlighted the critical need for early detection of HIV infection [46]. Gelaw YM, et al. found that the pooled prevalence of PJP in Ethiopia was 5.65% with high heterogeneity and they indicated that the frequency of PJP is probably underestimated, illustrating the variability in incidence rates in regions with limited access to healthcare [47]. Interestingly, a German study indicated that the incidence of PJP rose markedly from 2.3 to 2.6 cases per 100,000 population between 2014 and 2019, along with an increase in PJP-related deaths (from 516 to 615 cases) [48]. The risk populations traditionally receiving PJP chemoprophylaxis according to international guidelines (such as those with HIV, hematologic malignancies, or transplant recipients) experienced a significant decline in both cases and deaths, but individuals with solid tumors, autoimmune disorders, or pulmonary diseases showed a notable rise in incidence and mortality [48]. The data emphasize that the trend in PJP incidence has shifted, with a notable rise in mortality observed at the population level. Diagnosis of PJP relies on direct detection of the organism in respiratory samples, since clinical features and imaging are nonspecific [49]. Direct immunofluorescence offers higher sensitivity than conventional stains, and diagnostic yield increases from induced sputum (<50 to >90%) to bronchoalveolar lavage (90–99%), and lung biopsy (100%) [49]. Polymerase chain reaction (PCR), particularly quantitative PCR (qPCR), is increasingly used because of its high sensitivity and specificity, although it may not reliably distinguish colonization from active infection [49]. A higher fungal burden on qPCR correlates with clinically significant disease. The (1→3)-β-D-glucan assay may be a useful adjunctive diagnostic tool, as levels are often elevated in patients with PJP [49]. A low β-glucan concentration (e.g., <80 pg/mL using the Fungitell assay) makes PJP unlikely; however, specificity is limited because other fungal infections and certain medical interventions can also increase β-glucan levels [49].

Trimethoprim-sulfamethoxazole (TMP-SMX) remains the first-line treatment option for managing PJP in individuals with HIV [50]. While sulfa-based medications may cause resistance mutations in the *P. jirovecii* dihydropteroate synthase gene, such resistance rarely results in treatment failure [50].

## 5. *Cryptococcus neoformans* in Patients with HIV

Disseminated infection caused by *Cryptococcus neoformans* or *Cryptococcus gattii* is a severe opportunistic disease that typically develops in untreated HIV-infected individuals. In total, about 90% of cryptococcal infections in individuals with HIV occur in those whose CD4 T cell counts are below 100 cells/mm^3^ [51]. While the infection originates in the lungs, meningitis or meningoencephalitis are the most common clinical manifestations of cryptococcosis in patients with advanced immune suppression. Rajasingham R, et al. estimated that 4.3 million adults worldwide have CD4 T cell counts below 200 cells/mm^3^ and the mean global prevalence of cryptococcal antigenemia among HIV-infected individuals with CD4 counts under this threshold is 4.4%, corresponding to approximately 179,000 cases of cryptococcal infection globally in 2020 [52]. Each year, an estimated 152,000 cases of cryptococcal meningitis occur, leading to about 112,000 deaths attributable to cryptococcosis [52]. Overall, cryptococcal disease accounts for roughly 19% of all AIDS-related deaths worldwide [52]. The widespread use of antiretroviral therapy has led to a significant decline in the incidence of cryptococcal meningoencephalitis. In 2007, global estimates suggested roughly 957,900 annual cases of cryptococcal meningoencephalitis, leading to over 600,000 deaths and the highest disease burden was reported in sub-Saharan Africa (approximately 720,000 cases; range 144,000–1.3 million) [53]. In 2014, a subsequent analysis estimated 223,100 cases and 181,100 deaths per year globally, with cryptococcal infection accounting for about 15% of AIDS-related deaths worldwide [54]. Comparing data from 2007, 2014, and 2020, a decline in the number of cases and deaths due to cryptococcosis is observed. However, despite broader access to effective ART worldwide, the prevalence of cryptococcal disease remains persistently high in Africa due to limited access to medical care and largely unavailable recommended antifungal drugs (amphotericin B and flucytosine) [55]. Cryptococcosis is well-documented among individuals with HIV/AIDS, but it is now increasingly identified in other immunocompromised patients [56]. According to WHO recommendations, in adults, adolescents, and children living with HIV, who are suspected of having a first episode of cryptococcal meningitis, a prompt lumbar puncture with measurement of cerebrospinal fluid (CSF) opening pressure and a rapid cryptococcal antigen (CrAg) assay in parallel with CSF cryptococcal culture should be performed as the preferred diagnostic approach [57]. Where CrAg assays are unavailable or rapid testing is not feasible, CSF India ink examination remains the preferred alternative [57]. However, India ink has limited sensitivity; therefore, a negative India ink result should be verified using CSF cryptococcal antigen testing or a CSF culture. When lumbar puncture is unavailable or contraindicated, a cryptococcal antigen test performed on serum, plasma, or whole blood in parallel with blood culture is recommended as the initial diagnostic approach [57].

Treatment of cryptococcal infections remains limited. The standard therapeutic regimen includes amphotericin B and two synthetic agents, flucytosine and fluconazole. Both amphotericin B and flucytosine are toxic, and are often inaccessible in low-income regions due to their high cost [58]. In contrast, fluconazole is affordable, well-tolerated, and broadly available, yet its fungistatic nature has contributed to the emergence of resistant strains and frequent relapses in cryptococcal meningitis [58]. For most individuals with cryptococcal meningoencephalitis, combination of intravenous (IV) amphotericin B and oral flucytosine as the induction treatment is preferred [59]. Where resources allow, liposomal amphotericin B and flucytosine should be used for a minimum of two weeks [59]. When resources are limited, a single (10 mg/kg) dose of liposomal amphotericin B combined with 2 weeks of oral flucytosine and fluconazole is preferred [59]. Induction therapy should be used for minimum 2 weeks, and the course should be prolonged if the patient shows insufficient clinical improvement or if laboratory parameters have not yet been achieved (e.g., positive culture in individuals with CNS disease) [59]. After induction therapy is completed, patients should receive consolidation treatment with fluconazole for at least eight weeks [59]. Once induction and consolidation phases are completed, long-term maintenance therapy (minimum duration of 1 year) with a lower dose of fluconazole (200 mg daily) should be continued to prevent relapse [59]. Maintenance treatment may be withdrawn in individuals on ART whose CD4 cell count has reached ≥100 cells/µL and who have sustained an undetectable viral load for more than three months [59]. Consequently, there is a need for new or adjunctive therapeutic options to improve treatment outcomes.

## 6. Histoplasmosis in Patients with HIV

Histoplasmosis is a fungal infection caused by *Histoplasma capsulatum* that occurs endemically in certain regions, especially including the Mississippi and Ohio river valleys, the Caribbean, southern Mexico, and certain parts of Central and South America [60]. Moreover, this infection occurs in Asia, Africa, and Australia [60]. In healthy individuals, histoplasmosis is often asymptomatic or restricted to self-limited pulmonary infection [60]. In contrast, immunocompromised patients may develop a progressive disseminated form, which is considered an AIDS-defining condition [60]. Historically, due to limited awareness and inadequate diagnostic capabilities, the disease has been underrecognized, and its true global burden remains unclear [61]. Wheat LJ, et al. showed that at the beginning of the HIV epidemic histoplasmosis was a common opportunistic infection in patients with AIDS [62]. Current data suggest that although effective antiretroviral therapy reduces the risk of disease, histoplasmosis continues to be one of the most prevalent infections among people living with HIV in Latin America [63]. A systematic review published by Centers for Diseases Control and Prevention (CDC) underlined that in people living with HIV, histoplasmosis can be challenging to diagnose because the clinical manifestations of disseminated histoplasmosis closely resemble those of disseminated tuberculosis, which raises the risk of misdiagnosis and inadequate treatment, particularly in areas where both infections are common [61]. Although histopathology and culture offer the most reliable diagnostic approaches, many resource-limited settings lack the trained staff and laboratory facilities needed to perform these tests [61]. They showed that across 15 studies evaluating histoplasma antigen in the urine of 5096 people living with HIV, the overall prevalence was 11%, rising to 13% among those with advanced HIV, and in symptomatic individuals, prevalence reached 14%, while in asymptomatic patients it was 5% [61]. These results indicate that availability of rapid, point-of-care antigen tests would improve the assessment of histoplasmosis prevalence in resource-limited settings, where advanced HIV infection is common [61].

Prompt initiation of treatment in AIDS patients is essential because disseminated disease carries a high risk of death. Data showed that fluconazole is not as effective as itraconazole for *Histoplasma capsulatum* and induces resistance during therapy [64]. First-line treatment with itraconazole is recommended for HIV-positive patients who present with mild to moderate disease with nonmeningeal involvement [65]. In contrast, individuals with moderately severe or severe illness should receive an amphotericin B formulation as the initial therapy [65].

## 7. Other Selected Fungal Infections

Mucormycosis is a rare occurrence in individuals living with HIV, and most documented cases have other underlying risk factors. It remains uncertain whether the disease presents differently in HIV-positive patients depending on the presence of additional conditions such as neutropenia, diabetes, or a history of transplantation. Systematic review analyzed 94 cases of mucormycosis in people living with HIV and they were grouped into three categories: (1) HIV-infected individuals with other predisposing factors (*n* = 50), (2) intravenous drug users (*n* = 24), and (3) patients without any additional risk factors (*n* = 19) [66]. Among intravenous drug users, renal (41.7%) and cerebral (39.2%) involvement were the most frequent manifestations, while rhino-orbital disease was uncommon (4.2%) [66]. In contrast, rhino-orbital mucormycosis was the predominant form in the other two groups. The *Rhizopus* species were the most commonly isolated fungi overall, whereas *Lichtheimia* was most frequently identified in intravenous drug users [66]. The overall mortality rate was 53%, with no significant differences across the three categories [66]. These findings underscore that mucormycosis remains infrequent in HIV-positive individuals unless additional risk factors or intravenous drug use are present. *Mucorales*, *Fusarium*, *Scedosporium*, and other rare molds have intrinsic resistance to many drug classes, leading to difficult-to-treat infections.

Aspergillosis is no longer classified as an AIDS-defining condition, yet it continues to occur in many individuals with HIV/AIDS and is linked to substantial mortality [67]. Therefore, it should remain part of the differential diagnosis in HIV/AIDS patients who present with unexplained fever or pulmonary infiltrates, particularly when cavitary lesions are present.

## 8. Challenges in Diagnostics and Treatment of Fungal Infections

Major obstacles in fungal diagnostics include the time-consuming nature of conventional methods, low sensitivity, limited standardization of molecular tests, difficulty distinguishing colonization from infection, lack of reliable biomarkers, limited diagnostic techniques for deep-seated or CNS infections, access and resource limitations—advanced diagnostics are expensive and unavailable in many regions. Moreover, for some fungi, e.g., *Mucorales*, many labs lack identification capability, and delayed or incorrect identification leads to delayed therapy and worse clinical outcomes. A prompt diagnosis is essential to reduce the high morbidity and mortality linked to systemic fungal infections, as delayed detection almost leads to a poor outcome. This underscores the need for continued research, the standardization of existing techniques, and the boarded access to diagnostics tools in regions with limited resources. Therapeutic challenges include limited antifungal drug classes, rising antifungal resistance, toxicity and drug interactions, poor penetration into certain tissues (e.g., CNS, eyes), delayed initiation of therapy, and biofilm-associated infections. Antifungal stewardship is necessary to limit overuse or misuse of antifungals; furthermore, there is a need for new or adjunctive therapeutic options to improve treatment outcomes.

## 9. Conclusions

In well-developed settings, the frequency of fungal opportunistic infections in people living with HIV decreased due to the broad use of antiretroviral therapy. However, in untreated/abandoned patients with HIV or HIV-positive individuals with poor adherence to antiretroviral therapy, opportunistic infections may occur. Moreover, it is essential to emphasize the regional differences in the incidence of fungal infections. Still, invasive fungal infections are associated with high mortality. The challenge in antifungal treatment is the emergence of drug resistance.

Furthermore, environmental changes contribute to the emergence of new pathogenic fungi and to their geographic range expansion. Early diagnosis of HIV, starting antiretroviral therapy, and good adherence are still priorities. It is worth highlighting that the population at risk for fungal infections—including those with hematologic malignancies, transplant recipients, and patients receiving novel immunosuppressive therapies—is growing in the general population, making invasive fungal infections a concern not only for HIV-infected individuals.

## Figures and Tables

**Table 1:** Fungal priority pathogen list according to WHO [6].

Critical-Priority Group	High-Priority Group	Medium-Priority Group
*Cryptococcus neoformans*, *Candida auris*, *Aspergillus fumigatus*, *Candida albicans*	*Nakaseomyces glabrata* (*Candida glabrata*), *Histoplasma* spp., *Eumycetoma causative agents*, *Mucorales*, *Fusarium* spp., *Candida tropicalis*, *Candida parapsilosis*	*Scedosporium* spp., *Lomentospora prolificans*, *Coccidioides* spp., *Pichia kudriavzeveii* (*Candida krusei*), *Cryptococcus gattii*, *Talaromyces marneffei*, *Pneumocystis jirovecii*, *Paracoccidioides* spp.

## Data Availability

Not applicable.
